# Mass primaquine treatment to eliminate vivax malaria: lessons from the past

**DOI:** 10.1186/1475-2875-13-51

**Published:** 2014-02-07

**Authors:** Anatoly Kondrashin, Alla M Baranova, Elizabeth A Ashley, Judith Recht, Nicholas J White, Vladimir P Sergiev

**Affiliations:** 1 Sechenov First Moscow State Medical University, Moscow, Russia; 2 Mahidol-Oxford Tropical Medicine Research Unit (MORU), Faculty of Tropical Medicine, Mahidol University, Bangkok, Thailand; 3 Centre for Tropical Medicine, Nuffield Department of Medicine, Churchill Hospital, Headington, Oxford OX3 7FZ, UK

**Keywords:** Malaria, Elimination, Eradication, Mass drug administration, Primaquine, USSR, Glucose-6-phosphate dehydrogenase deficiency

## Abstract

Recent successes in malaria control have put malaria eradication back on the public health agenda. A significant obstacle to malaria elimination in Asia is the large burden of *Plasmodium vivax,* which is more difficult to eliminate than *Plasmodium falciparum*. Persistent *P. vivax* liver stages can be eliminated only by radical treatment with a ≥ seven-day course of an 8-aminoquinoline, with the attendant risk of acute haemolytic anaemia in individuals with glucose-6-phosphate dehydrogenase (G6PD) deficiency. Primaquine is the only generally available 8-aminoquinoline. Testing for G6PD deficiency is not widely available, and so whilst it is widely recommended, primaquine is often not prescribed. In the past, some countries aiming for vivax malaria eradication deployed mass treatments with primaquine on a massive scale, without G6PD testing. In Azerbaijan, Tajikistan (formerly USSR), North Afghanistan and DPR Korea 8,270,185 people received either a 14-day “standard” or a 17-day “interrupted” primaquine treatment to control post-eradication malaria epidemics. These mass primaquine preventive treatment campaigns were conducted by dedicated teams who administered the drugs under supervision and then monitored the population for adverse events. Despite estimated G6PD prevalences up to 38.7%, the reported frequency of severe adverse events related to primaquine was very low. This experience shows that with careful planning and implementation of mass treatment strategies using primaquine and adequate medical support to manage haemolytic toxicity, it is possible to achieve high population coverage, substantially reduce malaria transmission, and manage the risk of severe acute haemolytic anaemia in communities with a relatively high prevalence of G6PD deficiency safely.

## Background

Emboldened by recent successes in malaria control, malaria eradication has been put back on the public health agenda. The recent emergence of artemisinin resistance in Southeast Asia and pyrethroid resistance in Africa are additional powerful incentives to intensify malaria elimination efforts, both to prevent the spread of resistance across borders and to try to avoid a reversal of the recent substantial gains in malaria morbidity and mortality reduction [1]. A significant obstacle to malaria elimination in Asia is the high burden of vivax malaria since eradication will require elimination of the latent hypnozoite parasite stages that cause relapse. This requires radical treatment course of primaquine therapy, an 8-aminoquinoline anti-malarial drug, which causes dose-dependent acute haemolytic anaemia (AHA) of varying severity in individuals with glucose-6-phosphate dehydrogenase (G6PD) deficiency [2-4]. Primaquine is otherwise well tolerated if taken with food, although it may cause abdominal pain (ameliorated by co-administration with food), nausea, vomiting, occasional granulocytopaenia, and methaemoglobinaemia. The geographical distribution of G6PD deficiency, the most common inherited human enzymopathy [5], mirrors that of malaria because it provides some protection against the disease [6,7]. There are >180 different G6PD variants each associated with different degrees of deficiency. The more severe variants are prevalent in Southern Europe and Asia [2-4]. The risk and severity of AHA varies depending on the severity of the deficiency and the dose of primaquine given. G6PD deficiency testing is not widely available, hence there is understandable reluctance to prescribe primaquine to patients with unknown G6PD status in endemic areas.

The hypnozoitocidal activity of primaquine depends more on the total dose administered than on the length of the treatment [8]. Relapse patterns differ by geographic region; in temperate areas *Plasmodium vivax* typically relapses at an interval of eight to ten months whereas in most tropical regions the interval between relapses averages approximately three weeks [9]. For infections with long latency “strains” of *P. vivax* in temperate zones the standard course of radical treatment is 15 mg (base) adult dose daily for 14 days. In East Asia and Oceania a higher dose (30 mg base/day for 14 days) is needed to reduce relapse rates below 10%. In some countries of South Asia (e g, India, Nepal, Sri Lanka) the standard course of *P. vivax* treatment once consisted of five days, as estimates of *P. vivax* relapse rates there were low [10] but there is now general consensus that these short course, low dose treatments are ineffective. Primaquine is also recommended as an adjunctive treatment for falciparum malaria because of its unique gametocytocidal activity. Use of primaquine for both these indications is recommended by the World Health Organization (WHO) for all countries moving from control to elimination of malaria [11]. Recently, the WHO held a meeting to review a large body of evidence on the safety of primaquine [12] which, together with a meta-analysis of transmission blocking assessments, led to an official WHO recommendation of a lower dose of 0.25 mg/kg (instead of the previous 0.75 mg/kg) for use as a gametocytocide in falciparum malaria in low transmission areas without the need for G6PD testing [13]. Despite these recommendations, primaquine is often not used.

**Figure 1:**
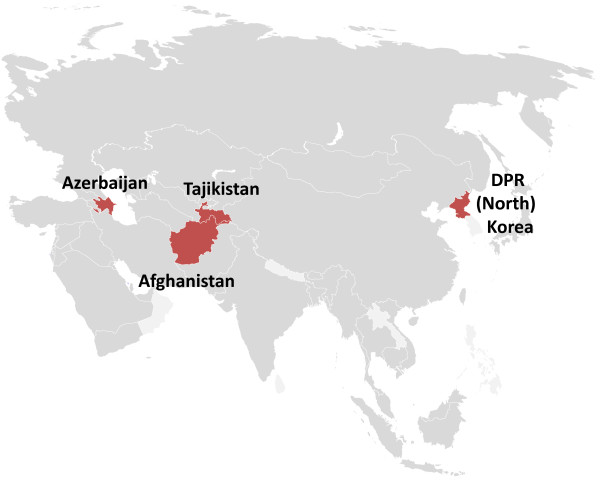
**MPPT sites.** The four countries (two were formerly Soviet republics) where MPPTs were conducted: Azerbaijan, Afghanistan, Tajikistan and DPR (North) Korea.

Mass drug administration (MDA) with anti-malarials has a chequered history, with some notable failures. For a period of time both chloroquine and pyrimethamine were added to cooking salt (Pinoti method) and resistance followed [14]. Conventional MDA with pyrimethamine also may have selected for resistance. Several different MDA strategies have been used, depending on the setting and whether *P. vivax* was also being targeted. Whichever strategy is used, high levels of population coverage are necessary for successful reduction or elimination of the transmission reservoir. The drugs used must have good acceptability, tolerability and safety profiles as most recipients are healthy. The 8-aminoquinolines have been used in MDAs since the first introduction of plasmoquine 90 years ago [15]. They are the only drugs which can clear dormant hypnozoites and thus prevent relapses of vivax and ovale malaria, but this benefit has to be balanced against the substantial risk of haemolytic toxicity in G6PD-deficient individuals. The first experience of the use of the USSR-synthesized quinocide (an 8-aminoquinoline drug which is a positional isomer of primaquine) as a MDA was conducted in 1955-56 involving several thousand people in the residual foci of *P. vivax* malaria in the Tadjik SSR (Lysenko, pers comm, 1971). This intervention, along with other anti-malaria measures, was considered instrumental in the arrest of local malaria transmission in the republic, and by the beginning of the 1960s all Tajikistan was free of locally acquired malaria. In most temperate areas *P. vivax* transmission is (or was) highly seasonal. Elimination of long-latency hypnozoites in infected persons during the low transmission season reduces *P. vivax* malaria transmission considerably during the next transmission season. This approach of seasonal chemoprevention with primaquine has been practiced until recently in temperate vivax endemic regions, e g, Central China and Turkmenistan, where the population residing in areas of active transmission received seasonal chemoprophylaxis with chloroquine and then after the season received radical treatment with 14 days of primaquine [16]. In a tropical setting, large-scale evaluations of primaquine (which succeeded plasmoquine in 1951) have been performed successfully in Nicaragua [17]. The 1970s experienced a series of “post-eradication malaria epidemics” in many parts of the world where malaria either had already been eradicated, or was very close to eradication [18]. Here the large MDA experiences (referred to henceforth as MPPT: “mass primaquine preventive treatment”) conducted in Azerbaijan, Northern Afghanistan, Tajikistan and DPR Korea are described (Figure 1) [19]. This MPPT resulted in substantial reductions of malaria incidence and proved safe in populations with varying degrees of G6PD deficiency. Although precise details are often unavailable from these large-scale implementations, this extensive experience contains important information of contemporary relevance. The results of the studies as described in official reports and contemporary accounts and the lessons learned from this MPPT experience are presented to inform and guide future implementation strategies.

### Azerbaijan

The first republic of the former USSR where a “post-eradication”, large-scale malaria epidemic occurred was Azerbaijan in 1969-71 [20]. Malaria had in fact never been eradicated completely and transmission continued to take place in a few localized foci. Nevertheless the major epidemiological features of the malaria epidemic in Azerbaijan corresponded to the WHO definition of a “post-eradication epidemic”. There were several relevant factors:

i) only one malaria species was involved (*P. vivax*)

ii) there appeared to be equal proportions of short and long incubation of *P. vivax* strains

iii) there was well-established resistance to DDT in the major malaria vector (*Anopheles maculipennis sacharovi*),

iv) there was markedly seasonal transmission

v) there was insufficient implementation of malaria control measures aggravated by poor supply of equipment, laboratory reagents, and anti-malarial drugs.

The strategy employed to contain the malaria epidemic in this region expeditiously was implementation of MPPT in active malaria foci of *P. vivax*[18,19]*.* The other available conventional anti-malaria measures were considered complementary. The assumption underlying this radical intervention was that primaquine intake by all the inhabitants of the malaria focus during 14 days either after or before the transmission season would eliminate all the hypnozoites in infected persons, thus removing the source of infection at the beginning of the next transmission season. The potential efficacy of the MPPT was predicted to be high providing that high coverage was achieved.

### MPPT organization and implementation

The MPPT implementation process consisted of three phases: i) preparatory; ii) mass distribution of primaquine; and, iii) “mopping up” operations. The preparatory phase was carried out by the local health workers, re-inforced by the workers from areas where malaria was absent, and supported by the participation of local people and local authorities. Malaria specialists from research institutions from other republics were mobilized. Sanitary activists from the Red Cross and Red Crescent societies were involved for the whole period of treatment. As a first step, detailed lists of all inhabitants of every village/settlement were prepared and made available to drug distributors and controllers. Information and education campaigns were conducted. One team of primaquine distributors (usually two to three persons) was responsible for treating 200-250 people residing in areas in close proximity to each other. A physician-controller was responsible for five to six such areas within one to two settlements. He or she was responsible for organizing and controlling the distribution of the drug, checking the names of recipients, the quality of treatment, and organizing the surveillance of side effects. Not all of the population received treatment: infants, pregnant women and patients with chronic liver or kidney disease were excluded.

The second phase consisted of drug distribution. It was found that the most appropriate time to start drug distribution was in the early morning (06.00-07.00). By noon drug distributors had covered about 75-80% of allotted persons. The distribution of drugs recommenced at 17.00 aiming to reach the absentees from the morning. The main principle of the second phase was strict adherence of the drug distributor to the rule stipulating that drug must be given only to the mouth of the treated person, to ensure each daily dose was received. Treatment of young children was administered by their mothers or by an older member of the family, but only in the presence of the drug distributor. Primaquine was given after taking some food with plenty of water. If treatment was missed for two to three consecutive days, the duration of the treatment was prolonged. If primaquine was missed for ≥ four consecutive days, treatment was restarted. The physician-controllers were engaged in daily surveillance of side effects. All emergency support facilities were at their disposal, including hospitalization and blood transfusion. Contingency plans, such as use of weekly primaquine in G6PD-deficient individuals were not required.

Treatment of targeted people who could not receive a full course of MPPT was the main task of the third phase. The proportion varied usually between 5 and 10%. Overall, because the campaign was well organized, the total number of treated people with a full course of primaquine treatment during the spring of 1971 was more than 67,000 persons comprising about 90% of the targeted population [19,20]. MPPT continued to play a leading role in the containment of the malaria epidemic in 1972 with 110,000 persons targeted and a coverage varying from 87 to 93%.

### Prevalence of G6PD deficiency in Azerbaijan

**Figure 2:**
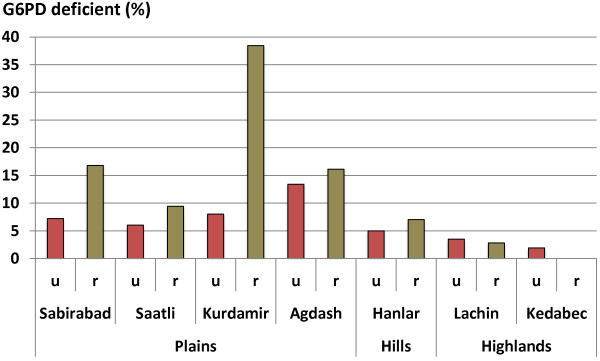
**Prevalence of G6PD deficiency in Azerbaijan in 1971-72.** Prevalence (%) of G6PD deficiency is shown for populations in the seven indicated districts: the first four were located in plains, one was in the foothills, and the remaining two in the highlands. In each district, proportions for urban areas are shown by red bars (u) and for rural areas by green bars (r). The number of people examined for G6PD deficiency ranged from 137-732 for urban areas, and 55-313 for rural areas.

The first data on the overall prevalence of G6PD deficiency in indigenous populations in the target area came from studies carried out in the Massali district [21]. Further investigations in Barda and Lenkoran districts revealed a prevalence of 7% when assessed by the Berstein method [22]. During 1971-73 a total of 24,000 inhabitants in 77 settlements of 18 districts of Azerbaijan were screened [23]. The frequency of G6PD deficiency varied widely from place to place (Table 1; Figure 2).

**Table 1:** Prevalence of G6PD deficiency in Azerbaijan in 1971-72

**District**	**Landscape**	**Type of population**	**N examined**	**Prevalence (%)**
Sabirabad	Plains	Urban	534	7.2
		Rural	225	16.8
Saatli	Plains	Urban	400	6.0
		Rural	106	9.4
Kurdamir	Plains	Urban	315	8.0
		Rural	55	38.4
Agdash	Plains	Urban	365	13.4
		Rural	212	16.1
Lachin	Highlands	Urban	732	3.5
		Rural	144	2.8
Hanlar	Hills	Urban	457	5.0
		Rural	313	7.0
Kedabec	Highlands	Urban	137	1.9
		Rural	153	0.0

**Figure 3:**
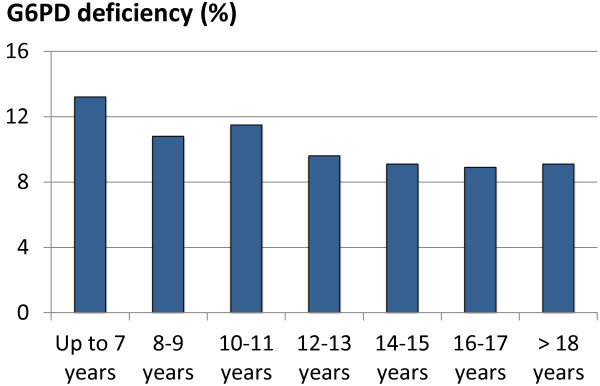
**Prevalence of G6PD deficiency by age in Azerbaijan in 1971-72.** Prevalence (%) of G6PD deficiency is shown for the different age groups. For each group, a total of between 909 and 3,083 people were tested by the Berstein method.

The prevalence of G6PD deficiency assessed by the Berstein method [22] ranged from 0% among Russian resettlers and local people of the highlands, to 38.4% among the indigenous inhabitants of the Kura-Araks plains. A striking difference was observed between the urban and rural populations; the prevalence was higher among inhabitants of rural areas in the plains compared with that in urban populations [23]. The opposite relationship was observed among the inhabitants of the highlands, where the prevalence of G6PD deficiency was higher in urban areas probably because of migration of people from the plains. The G6PD deficiency prevalence did not vary significantly among various age groups suggesting no major mortality advantage or disadvantage after birth (Table 2; Figure 3).

**Table 2:** Prevalence of G6PD deficiency by age in Azerbaijan in 1971-72

**Age group**	**N examined**	**Prevalence (%)**
Up to 7 years	909	13.2
8-9 years	2347	10.8
10-11 years	3083	11.5
12-13 years	2899	9.6
14-15 years	2161	9.1
16-17 years	1527	8.9
> 18 years	1049	9.1

### A modified “interrupted” MPPT scheme for populations with a high prevalence of G6PD deficiency

The target primaquine dose was an adult dose of 210 mg (base) corresponding to approximately 3.5 mg base/kg. In order to reduce or prevent possible side effects in the treated population during the MPPT in 1971, given the high prevalence of G6PD deficiency in certain areas, a putatively “safe” scheme of MPPT was developed [24] based on two known facts: i) primaquine-related haemolytic manifestations are typically delayed with respect to drug administration as residual erythrocyte anti-oxidant defences are exhausted; and, ii) primaquine-induced haemolysis removes the “older” population of erythrocytes with the lowest content of G6PD [2-4]; reticulocytes replacing haemolysed erythrocytes have greater enzyme content and so are considerably more resistant to the haemolytic action of primaquine. The new “safer” scheme was called “interrupted” and consisted of a regimen of primaquine given for four days, stopped on days 5-7, and then restarted on day 8 continuing through to day 17.

This “interrupted” MPPT was originally implemented in the Geokchai district in Azerbaijan in 1971 because there was a high prevalence of G6PD deficiency compared with other malaria-affected districts. Overall the prevalence of G6PD deficiency was 15.4% (Figure 3), while in three specific villages it varied from 31.2 to 38.7%. *Plasmodium vivax* malaria incidence was also one of the highest in the republic. It was expected that the incidence of side effects would be around 10%. Therefore the MPPT was carried out with great caution; infants and pregnant and lactating mothers were excluded. The MPPT was carried out in phases: initially MPPT was conducted in eight villages, followed by treatment in the next 20 villages, and finally in the remaining eight villages. There was excellent organization and medical supervision. All medical staff engaged in the surveillance activities were provided with the names of individuals with G6PD deficiency previously detected by selective surveys, which enabled them to monitor closely the status of their health during the whole course of the treatment. A number of clinicians with experience in haematology conducted daily visits on the spot, supported by laboratory personnel ready to carry out emergency examinations.

The MPPT was administered to 30,000 persons in total, with the prior expectation that side-effects would be reported in around 3,000-4,000. Unexpectedly, an overwhelming majority of treated people did not experience any serious side effects due to primaquine. Overall an estimated 1% complained of adverse effects with G6PD-deficient individuals reporting mild or moderate side effects like dizziness, headache, back pain, change of urine colour, mild scleral icterus. All these symptoms appeared three to six days following the start of treatment and importantly they did not progress further in spite of continuation of the treatment. Individuals who complained of allergic reactions including skin rash after taking primaquine continued the drug whilst taking antihistamine drugs. The results of laboratory examination of cases complaining of side effects in some instances revealed the presence of other diseases and causes not related to primaquine treatment. A few persons with G6PD deficiency had more serious complications including severe headache, back pain, fatigue, red or black urine, and jaundice. Primaquine administration was discontinued and supportive treatment was administered which resulted in rapid recovery. Remarkably, out of 30,000 treated people, only seven persons were hospitalized, and blood transfusion was not needed in any of these patients [24]. Stopping primaquine resulted in rapid normalization of laboratory indices. Overall persons without G6PD deficiency showed a reduction of haemoglobin up to 1-2 g/dL, and persons with G6PD deficiency had a reduction of up to 3-5 g/dL. However the level of haemoglobin usually did not decrease to below 9 g/dL. The decrease in haemoglobin was accompanied by reticulocytosis [25] and was reportedly more pronounced in children younger than 11 years.

### Epidemiological evidence of MPPT efficacy in Azerbaijan

**Figure 4:**
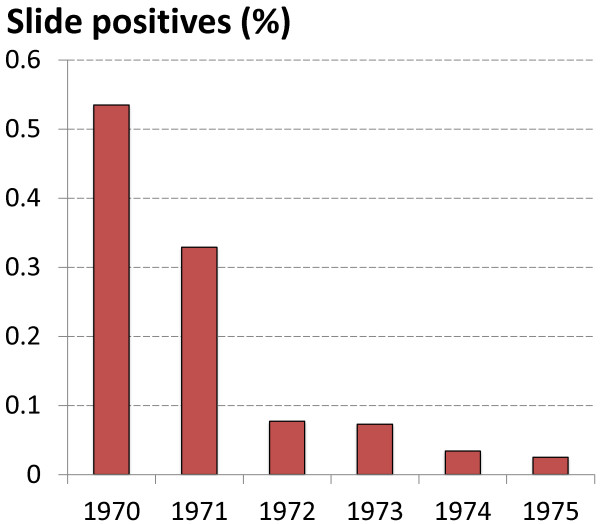
**Epidemiological assessment of efficacy of MPPT in Azerbaijan in 1970-1975.** Malaria cases as assessed by slide positivity rates (*P. vivax* detection from total slides examined, %) is shown for each year from 1970-75. The total number of slides examined was over one million annually, varying between 1,026,834 and 1,212,864.

**Figure 5:**
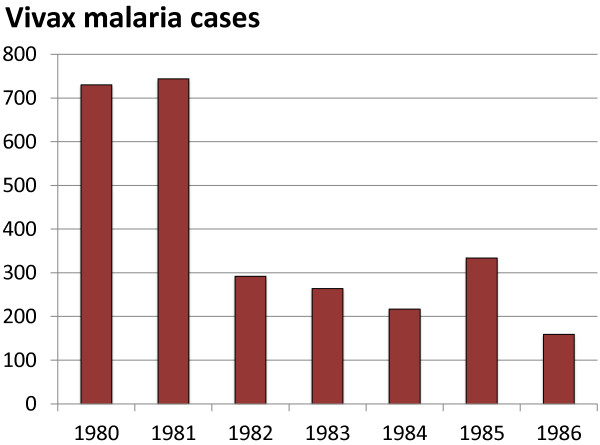
**Results of the MPPT in Azerbaijan in 1980-86.** MPPT was given to between 23,541 and 75,664 people annually between 1980 and 1986 resulting in a substantial reduction in the total number of malaria cases.

As shown in Table 3 (Figure 4), MPPT was one of the most important factors in the containment and control of a large-scale *P. vivax* epidemic in Azerbaijan in 1970-72 [26]. As a result of MPPT during 1971-75, the malaria epidemic was controlled and only a few residual malaria foci remained. Inadequate malaria control activities in the following years led to a deterioration of the malaria situation in Azerbaijan, and by 1979, malaria incidence had risen alarmingly (Table 4). Inadequacy of supply, equipment and insecticide during this time precluded large-scale use of vector control measures, leaving the health authorities with MPPT as the only available option to interrupt the malaria epidemic. As it had worked well previously, MPPT was therefore implemented again between 1980 and 1986 (Table 5; Figure 5). By 1982 the incidence of malaria showed a 60% reduction compared to the beginning of MPPT implementation, however there was no significant further decrease in malaria cases in subsequent years.

**Table 3:** Epidemiological assessment of the efficacy of MPPT in Azerbaijan in 1970-1975

**Year**	**MPPT (N)**	**Blood slide examination (N)**	**Number of positive slides**	**Slide positive rate (%)**
1970	-	1,131,589	6,055	0.535
1971	106,555	1,212,864	3,991	0.329
1972	61,994	1,034,679	800	0.077
1973	21,694	1,090,361	795	0.073
1974	31,726	1,026,834	352	0.034
1975	10,587	1,027,811	257	0.025

**Table 4:** Malaria incidence in Azerbaijan in 1979-81

**Year**	**Number of active foci**	**Total number of cases**
1979	146	165
1980	626	730
1981	313	744

**Table 5:** Results of the MPPT in Azerbaijan in 1980-86

**Year**	**MPPT (N)**	**Number of cases**
1980	35,045	730
1981	43,967	744
1982	75,664	292
1983	51,000	264
1984	53,087	217
1985	23,541	334
1986	65,924	159

### Comparative efficacy of different MPPT administration schedules

The efficacy of the MPPT “standard” course was compared with the “interrupted” course in Kendoba village of the Aksu district and in four villages of the Geokchai district [27] (Table 6), all areas with high malaria incidence. For both schedules MPPT coverage was high and efficacy excellent.

**Table 6:** Efficacy of standard and “interrupted” MPPT courses; Azerbaijan

	**Standard course**	**“Interrupted” course**
Total population targeted	934	5,815
MPPT coverage	878 (94%)	5,316 (91.4%)
Number of cases during malaria transmission season	7 (0.8%)	21 (0.4%)

A total of 2,972 people were treated with the standard course of MPPT in two *P. vivax* malaria-endemic districts (Zhdanov and Mir-Bashir) where the prevalence of G6PD deficiency varied from 5 to 10% [28]. Infants, pregnant and lactating mothers, and persons with chronic diseases were excluded. A total of 79 side effects were recorded (on average 2.66%, range 0.86 to 4%) with no hospitalization required and only a few people stopped treatment before completing their drug course. These results are in good agreement with the earlier experience [24].

### Clinical and diagnostic aspects of G6PD deficiency

The average prevalence of G6PD deficiency in persons not infected by *P. vivax* (as determined by absence of parasites assessed by microscopy examination of blood samples) was 15.2%, while among patients presenting with vivax malaria it was 3.6% [25]. This large difference suggests that G6PD deficiency, although it does not protect from acquiring malaria infection, may attenuate the clinical manifestations of *P. vivax* malaria. Clinical MPPT investigations revealed that malaria patients with G6PD deficiency tolerated primaquine much better than non-infected persons with the deficiency. The investigators [25] thought this might be explained by the malaria infection itself causing anaemia, with the preferential destruction of “old” erythrocytes, so in G6PD deficiency the proportion of vulnerable erythrocytes would be lower in malaria than in healthy subjects. Clinically significant adverse events observed following the daily 15 mg dose during the MPPT consisted of symptoms and signs of moderate haemolysis and anaemia which were associated with G6PD deficiency [29]. Most noticeable side effects were recorded in patients with very low activity of the G6PD enzyme. Concomitant viral or bacterial infections were observed to exacerbate haemolysis. Overall, clinicians concluded that the presence of G6PD deficiency did not preclude the implementation of MPPT, providing strict daily medical supervision was available during the campaign.

### Afghanistan

Based on the MPPT experience accumulated in Azerbaijan during 1971-72, the same strategy was applied successfully to the *P. vivax* malaria foci in the north of Afghanistan in 1972-73 on the basis of an agreement between the Governments of Afghanistan and the USSR. The decision to carry out MPPT was prompted by the poor efficacy of other malaria control measures implemented, particularly vector control activities [30]. Malaria control activities were planned jointly, organized and implemented by the local health services, personnel of the Martzinovski Institute and the staff of the Kabul Institute of Malariology. Local authorities assisted in mobilizing sanitary activists for drug distribution.

Initially, as a field trial, the MPPT was carried out in one settlement, Kuldoman village, Kunduz province in November of 1972. A total of 1,937 persons of Uzbek ethnicity were treated with the standard 14-day regimen. A similar village, Hatankuli, was selected as a non-MPPT control. Absence of any serious side effects among the treated population served as a basis for the decision to expand the size of the targeted population. In December of 1972 MPPT was conducted in 14 villages in the same province with a total population of 14,028. The MPPT coverage exceeded 90%. The population of another 14 villages served as non-MPPT controls. Remarkably, no side effects were reported. Efficacy of the MPPT was very high, as the incidence of malaria in the treated population was reduced three-fold compared with the untreated population during the following malaria transmission season [30].

#### Prevalence of G6PD deficiency in North Afghanistan

**Figure 6:**
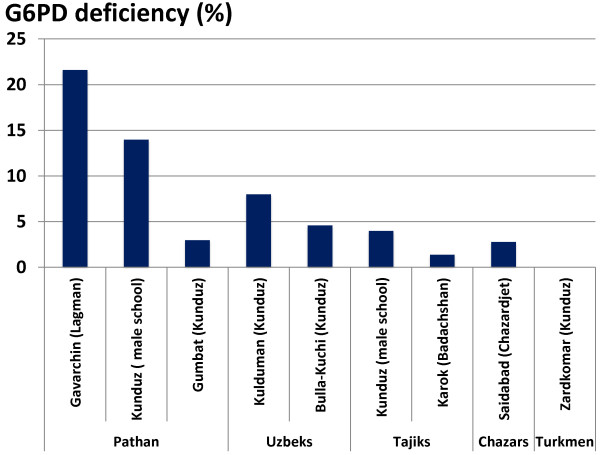
**Prevalence of G6PD deficiency in indigenous ethnic groups in North Afghanistan.** Between 50 and 175 people in each village (province indicated in parentheses) were tested for G6PD deficiency, the resulting prevalences (%) are shown. Results for Gavarchin, Bulla-Kuchi, Karok and Saidabad are from [24], and for Kunduz, Gumbat, Kulduman and Zardkomar from [25]. Villages are shown grouped by ethnicity: Pashtuns, Uzbeks, Tajiks, Chazars and Turkmen.

Before further expansion of MPPT, the prevalence of G6PD deficiency among the indigenous population of North Afghanistan was assessed (Table 7; Figure 6). Earlier investigations on the prevalence of G6PD deficiency had been conducted in the Kunduz province among Afghani Uzbeks [31] among whom the prevalence of G6PD deficiency was found to be 4.6%. In 1973 joint Afghan-Soviet studies were carried out in North Afghanistan in various local ethnic groups [32]. These data are similar to those published by WHO in 1968 from Israel on Afghani refugees; the Afghani Pashtuns (Pathan) appear to be more affected by G6PD deficiency than other ethnic groups. The G6PD variant most common in Afghanistan is the Mediterranean type which has been associated with the most severe deficiency [33].

**Table 7:** Prevalence of G6PD deficiency in indigenous ethnic groups in North Afghanistan

**Village (province)**	**Ethnic group**	**No. examined**	**G6PD deficiency prevalence %**	**Source**
Gavarchin (Lagman)	Pathan	116	21.6	[24]
Kunduz ( male school)	Pathan	50	14.0	[25]
Gumbat (Kunduz)	Pathan	100	3.0	[25]
Kulduman (Kunduz)	Uzbeks	100	8.0	[25]
Bulla-Kuchi (Kunduz)	Uzbeks	175	4.6	[24]
Kunduz (male school)	Tadjiks	100	4.0	[25]
Karok (Badachshan)	Tadjiks	139	1.4	[24]
Saidabad (Chazardjet)	Chazars	107	2.8	[24]
Zardkomar (Kunduz)	Turkmen	100	0.0	[25]

In 1973-74 a total of 78,000 were given MPPT in Kunduz province. The rate of side effects among the treated population was less than 1%, with the main symptoms being fatigue, headache, back pain, disorders of the alimentary tract, and change of urine colour. Two women reported passing black urine four to five days after starting treatment. However they did not require hospitalization.

### Tajikistan

#### Prevalence of G6PD deficiency in Tajikistan

The first data on G6PD deficiency among the indigenous population in Tajikistan were published in the 1980s [22]. Three different G6PD variants were identified: Dushanbe I and II, which showed less than 10% of residual enzyme activity, and Dushanbe III with normal enzyme activity [34]. More recent studies in Central Asia demonstrated a broad range in the frequency of the trait among the different ethnic groups: 2.1% among Iraqis, 2.9% in Pakistani Tajiks, and over 15.8% among Pakistani Pashtuns [35]. These data are very similar those shown in Table 7 for North Afghanistan. Studies were carried out in three regions of Tajikstan namely in Dushanbe, Dangara and Kabadiyon [35]. The overall prevalence of G6PD defiency was 2.1%, varying from 0.8% in Dangara to 1.6% in Dushanbe and to 4% in Kabadiyon [36].

#### MPPT in Tajikistan

Malaria was eradicated in Tajikistan at the beginning of the 1960s. Re-establishment of local malaria transmission occurred during the 1980s initially in border areas close to highly malarious villages in neighbouring Afghanistan across the Punj river. In order to reduce the case load in the newly established active *P. vivax* malaria foci, a total of 80,000 people received MPPT during 1983-85 [37]. However, a high coverage was not achieved (actual coverage was only 77%), which resulted in only a modest reduction of malaria incidence in the treated population. As a result, active malaria transmission continued in border areas.

Civil war in Tajikistan during the 1990s led to the exodus of more than one million Tajik refugees into the highly malarious areas of Afghanistan, where many were infected with malaria. Subsequent large-scale return of these infected refugees to their homeland devastated by the civil war favoured the establishment of numerous malaria foci in the absence of adequate malaria control and prevention measures. Implementation of malaria control measures on a national scale was resumed only at the end of the 1990s/early 2000s with assistance from bilateral and international agencies. The difficulties in controlling malaria made MPPT the only option available at the time to contain the malaria epidemic.

**Figure 7:**
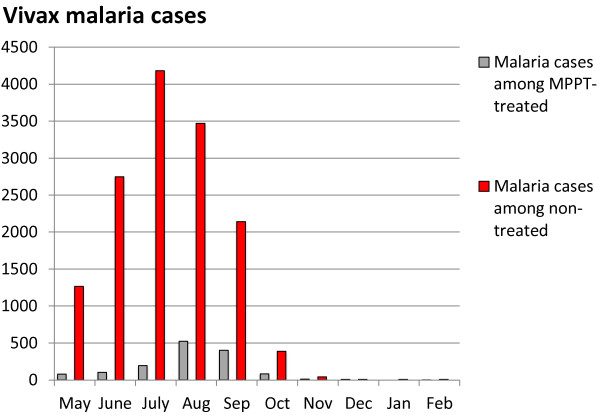
**MPPT in Tajikistan in 2000.** Reduction of malaria cases in two districts where MPPT was implemented is shown; 39,590 people were treated in Bachtar and 5,569 in Kofarnichon.

In 1998-1999, a total of 521,000 people from 12 southern districts of the republic were treated with primaquine. Although authorities in charge of the organization and implementation of the MPPT claimed that the number of reported side effects was very low, hard data were not made available. Nevertheless, the overall malaria situation in the republic shows a well-established declining trend, which, among other factors, may be attributed to the use of the MPPT even considering its limitations such as the quality of its implementation, particularly coverage (Precise data available only for 2000; Table 8: Figure 7).

**Table 8:** MPPT in Tajikistan in 2000

**District**	**Treated people**	**Malaria cases**	
		**Previous transmission season**	**2000 transmission season**
Bachtar	39,590	625	411
Kofarnichon	5,569	2,380	401

In 2002 a total of 287,081 people in 20 districts and towns were subjected to MPPT, which reduced the malaria incidence in Tajikistan by about 40% [38].

### The Democratic People’s Republic of Korea

Malaria in DPR Korea is caused exclusively by long latency *P. vivax*, and was considered eradicated at the beginning of the 1970s. The first indications of a probable deterioration of the malaria situation appeared at the end of the 1990s, particularly in 1998, when about 2,500 indigenous vivax malaria cases were reported officially in the southern border areas of the country. The peak occurred in 2001, when the total number of malaria cases was estimated at about 300,000. The resurgence of malaria is believed to be linked to the economic crisis during the 1990s, aggravated by a series of natural disasters resulting in a deterioration of the living conditions of the population. In addition, technical inadequacy in detecting re-emerging malaria in the country, along with a severe lack of anti-malarial drugs, particularly primaquine, laboratory equipment, reagents, and trained personnel prompted the government to appeal to WHO for technical and material assistance. WHO assisted the country through the development of an appropriate strategy to contain the malaria epidemic and mobilization of resources from international and bilateral agencies. In the absence of other means, MPPT was considered as the only affordable intervention that could check further proliferation of malaria through the rest of the country and reduce the burden of the disease in this impoverished population.

#### Epidemiological features of *Plasmodium vivax* malaria in DPR Korea

The season of malaria transmission in the Korean peninsula is relatively short, from the end of May or beginning of June through September, with the peak incidence during the months of July and August. One of the main features of epidemiology of malaria in the country was the presence of long latency *P. vivax* which exhibits both short- and long-incubation periods. Thus it was difficult to distinguish between primary manifestations and relapses. It was estimated that at the peak of the malaria epidemic, primary infections and relapses contributed equally to malaria infections. When untreated (without primaquine), an average relapse rate was about 40% with the number of malaria episodes averaging two to three per patient.

The age and sex distribution of malaria incidence reflects an occupation-related disease, confined to farmers (55% of all cases) and industrial workers (25%) with the remainder being office workers and students. With a slight preponderance of malaria in males (52%), children between 0-16 years constituted only 22% of the overall malaria incidence. A probable explanation of the high malaria incidence among farmers and industrial workers is that both groups are heavily exposed to bites of *Anopheles sinensis,* the main malaria vector in the country, during agricultural work in the paddy fields.

#### MPPT Implementation in the DPR Korea

A well-developed primary health care system, the backbone of which is the institution of the Section (Household) Doctor assigned with the task of serving around 600-700 persons and assisted by the network of Health Volunteers at village level, prompted the Ministry of Health to endorse the WHO proposal to deploy MPPT. It was decided that the deployment of MPPT would be undertaken during the pre-transmission season (April-May) in order to prevent the occurrence of primary manifestations of long incubation period infections and to prevent long latency relapses, thus substantially reducing the source of infection just before the malaria transmission season. The decision to deploy the MPPT was strongly influenced by the encouraging results obtained by DPR Korea in the past with primaquine, and by experiences of other countries with similar *P. vivax* using MPPT, notably by neighbouring China. Over the past 40 years China has used MDAs with regimens including primaquine to millions of people [39], notably in Henan province, where a total of 803,000 people were treated during the 1990s [40].

The DPR Korean National Malaria Control Programme identified the population of MPPT target areas. Some Ri (a term used to describe a group of eight to 11 villages) were under the intervention, while other neighbouring Ri were not, thus allowing evaluation of the efficacy of the mass treatment. Necessary health education activities among the targeted population were carried out including explanation of the purpose of MPPT and the importance of cooperation and reporting side effects. MPPT organization was under the Directors of County Hygiene and Anti Epidemic Stations. The personnel of the County and Ri clinic/hospitals under their Directors, Household Doctors, and Health Volunteers were all involved in primaquine distribution. The size of the allotted population for each team varied from place to place, but were on average: one to two Household Doctors, one to two senior health workers together with five to ten health volunteers for approximately 500-600 people.

Primaquine 15 mg a day (adult dose) during 14 days was administered at an appointed place every morning after breakfast with a glass of water directly to the mouth. Daily supervision of the drug distribution was carried out by the personnel of the Provincial and County Health institutions. Children younger than five years and pregnant women were excluded from the MPPT. Drug recipients were interviewed daily about side effects during the previous day. With the involvement of additional staff for drug distribution provided by the People’s Committees, it became possible to visit people at their homes, thus further increasing the coverage and the efficacy of the treatment. Even though G6PD deficiency prevalence in DPR Korea was estimated at 0.5-2.9% by WHO in 1989 [41], which is considered low, precautions were taken to deal with any serious side effects during MPPT. The staff of the County and Ri hospitals were instructed on possible side effects and a special form was developed and made available to health workers containing management advice which was to be used for case documentation.

### MPPT epidemiological efficacy

**Figure 8:**
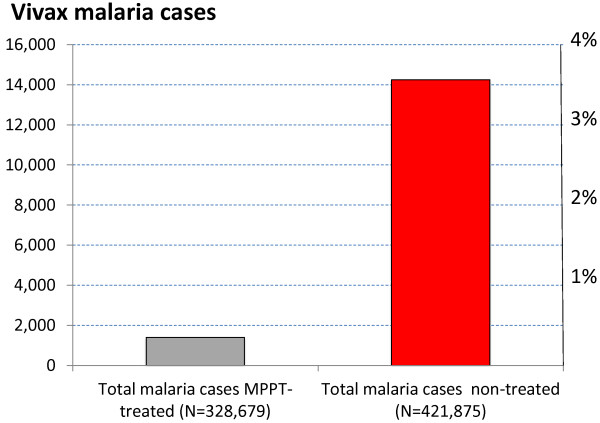
**Comparing vivax malaria (cases) among the treated and untreated populations, 2002-2003; DPR Korea.** From a total of 328,679 MPPT-treated people and 421,875 who were untreated, total cases of malaria and percentages are shown.

Following MPPT in 2002 (started on 18 April and completed on 3 May, 2002) two efficacy assessment methods were deployed. The first (Table 9; Figure 8) consisted of a direct evaluation of malaria incidence in several Ri of eight counties under the intervention (with a total of 328,679 treated population) compared with the malaria incidence among non-treated in the same counties (a total of 421,875). The malaria incidence (overall for ten months) was 0.426% in treated people *versus* 3.38% among untreated people.

**Table 9:** Comparative malaria (cases) among treated and untreated population, 2002-2003; DPR Korea

**Population**	**May**	**June**	**July**	**Aug**	**Sep**	**Oct**	**Nov**	**Dec**	**Jan**	**Feb**	**Total (%)**
Treated (total 328,679)	79	104	193	523	402	82	10	8	0	1	**1,402 (0.426)**
Non treated (total 421,875)	1,264	2,747	4,182	3,470	2,142	388	42	8	6	7	**14,256 (3.38)**

**Figure 9:**
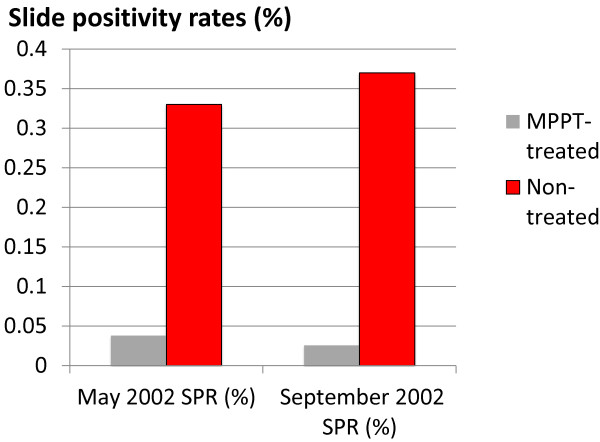
**Slide positivity rates (%) in the treated and untreated population, 2002.** DPR Korea. Slide positive rates (%) are shown for treated (N = 5,138) and untreated (N = 4,215) populations in May and September 2002.

The second approach was a blood survey among those who received MPPT and those who did not which was conducted in May 2002 just before the malaria transmission season, and in September 2002 at the end of it (Table 10; Figure 9). Together, these results illustrate the major role of MPPT in reducing malaria incidence in the counties where it was implemented.

**Table 10:** Slide positive rates (%) in treated and untreated population, 2002: DPR Korea

**Population**	**MAY 2002**			**SEPTEMBER 2002**		
	**Slide examined**	**Slide + ve**	**SPR %**	**Slide examined**	**Slide + ve**	**SPR %**
Treated	5,138	2	0.038	3 716	1	0.026
Non treated	4,215	14	0. 33	2 994	11	0.37

**Figure 10:**
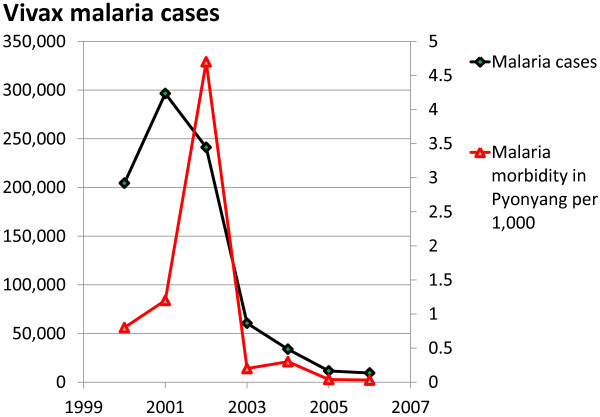
**MPPT impact in 2000-2006.** DPR Korea. Total number of malaria cases are shown for the years 2000-2006 (black line, left Y axis) and malaria morbidity in the capital Pyonyang per 1,000 (redline, right Y axis).

Encouraged by these very impressive results, MPPT was expanded to all areas with high and moderate malaria transmission (Table 11). Available data revealed a high MPPT coverage ranging from 94% in 2003 to 98% in 2006, one exception being the low coverage in 2004 (78%). The latter was attributed to the fact that MPPT was given also to the population of Kaesong city in 2004, where the proportion of people excluded from the treatment was higher than elsewhere, as was the number of absentees. There was a considerable reduction of malaria incidence in all areas under MPPT during 2002 compared to 2001, followed by an even more pronounced reduction in 2003 (by 72%). This trend was maintained in the following years as well (Table 12; Figure 10). The year 2002 was considered as baseline, when MPPT commenced in selective areas with high and moderate malaria transmission levels (Table 13). A good supportive indicator of a genuine reduction of malaria case load in the country is the dynamics of malaria morbidity among imported cases in the capital Pyonyang, where local transmission could be excluded; there was a pronounced reduction starting in 2003 (Table 12).

**Table 11:** **MPPT in areas of DPR Korea with high and moderate *Plasmodium vivax *** **malaria transmission in 2002-2007**

**Province**	**Population**	**Population treated D**						
		**2002**	**2003**	**2004**	**2005**	**2006**	**2007**	**Total**
S.Hwangae	2,274,642	74,160	425,475	123,162	33,831	-	2,054,980	2,711,608
N.Hwangae	2,076,473	99,000	-	269,667	88,001	37,8366	1,814,043	2,649,107
Kangwon	1,446,256	43,345	-	-	65,095	-	1,035,238	1,143,678
S.Pyongan	3,977,179	84,354	-	-	113,595	-	-	197,949
N. Pyongan	2,689,779	64,000	-	-	149,178	-	-	213,178
Pyongyang*	-	31,787*	-	-	-	-	-	31,787
Total	12,464,329	396,646	425,475	392,829	449,700	378,366	4,904,261	6,947,277

*Local transmission was not possible in this province. All treated cases were considered as imported with infection acquired outside the city.

**Table 12:** MPPT Impact in 2000-2006; DPR Korea

**Year**	**2000**	**2001**	**2002**	**2003**	**2004**	**2005**	**2006**
Malaria cases	204,428	296,540	241,190	60,559	33,803	11,507	9,353
Pyonyang malaria morbidity (per 1,000)	0.8	1.2	4.7	0.2	0.3	0.04	0.03

**Table 13:** Malaria among individuals excluded from MPPT in 2002; DPR Korea

**County/Ri**	**MPPT target population**	**Total excluded**	**Malaria cases among excluded**	**Morbidity (%)**
Panmun	30,000	2,100	18	0.86
Hwanju	74,030	10,000	147	1.47
Anbyon	40,970	4,978	123	2.47
Sonchon	54,470	6,792	45	0.66
Kangnam	33,030	1,950	14	0.72
Sukchon	86,000	10,133	114	1.12
Hwanhu Ri	2,263	542	18	3.32
Total	320,763	36,495	479	1.31

### MPPT safety

As a first step to prevent possible side-effects, children < five years of age and pregnant women were excluded. In 2002, at the onset of the MPPT, a total of 391,357 people from 91 Ri of seven malaria high-risk counties had been identified for MPPT. A total of 57,411 persons were excluded from treatment on various grounds. A total of 5,267 persons could not complete a full course of primaquine treatment (1.6%) for various reasons including duty travel and other absences.

**Figure 11:**
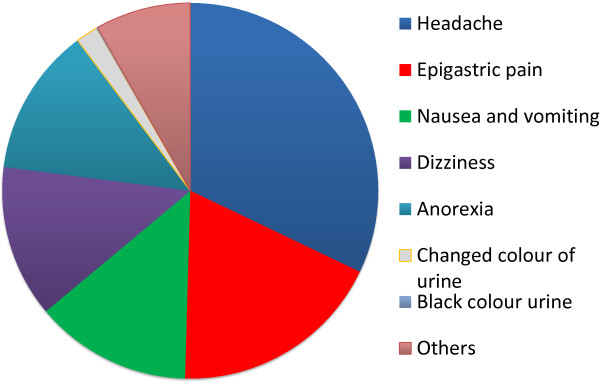
**Prevalence of side effects during MPPT in 2002.** DPR Korea. Prevalence (%) reported for MPPT in 2002 is indicated for each side effect. There was a total of 4,291 headache cases, 2,451 epigastric pain, 1,801 nausea and vomiting, 1,753 dizziness, 1,702 anorexia, 254 change in urine colour (1.9%), and 15 black urine (0.1%).

Nearly 400,000 follow-up cards containing pertinent questions about the occurrence of side effects were printed in Korean and distributed to all drug distributors and controllers. Analysis of the content of these cards revealed that the frequency of various side effects did not exceed 4%. Most importantly, no cases of severe haemolysis were reported (Figure 11). Fifteen cases of black urine suspected to be due to haemolysis fully recovered within three to four days after primaquine administration was stopped without the need for hospitalization. Women complained of side effects twice as much as men, except for black urine. Prevalence of side effects was lowest in children up to 16 years of age. Prevalence of side effects among the treated population in the following years was even lower, not exceeding 1.5% in 2003 and 2004 [42].

### MPPT limitations

The use of MPPT during five consecutive years (2002-2006) resulted in a significant reduction of malaria morbidity in DPR Korea. However, malaria transmission could not be completely eliminated even in areas with the highest reduction of incidence in the absence of complementary malaria control measures, particularly vector control. Only concomitant use of MPPT and vector control activities will result in the interruption of local malaria transmission as was successfully demonstrated during the final stages of the malaria eradication program in Tajikistan at the end of the 1950s.

The highest efficacy of MPPT is achieved at coverages of more than 90%. Obtaining 100% coverage is impossible because some populations (young children, pregnant women, patients with some chronic diseases, absentees, etc) are excluded from MPPT and may harbour malaria parasites and serve as an ongoing source of infection.

## Discussion

MDA to eliminate malaria has had a chequered history [14], but one that is being re-examined now as malaria elimination is now firmly back on the global agenda. The resurgence of interest in malaria has focused naturally on high burden areas with high transmission of falciparum malaria, and a high morbidity and mortality burden. Considerable scale-up in the deployment of effective vector control interventions (notably insecticide-treated bed-nets) and effective drugs (ACT) has reduced this burden substantially. Belated recognition of the importance of vivax malaria in Asia and the Americas has focused primarily on areas of higher transmission in tropical areas, and the long latency vivax malaria prevalent in temperate areas has been all but forgotten. Vivax malaria is more difficult to eliminate than falciparum malaria, primarily because of relapse. Elimination of vivax malaria is therefore not possible in a short time frame without tackling the persistent liver stages. This requires use of primaquine with the attendant risk of haemolytic toxicity in subjects who are G6PD deficient. Few of the current generation of malariologists have any first-hand experience of MDA with effective drugs. The extensive MDA experience with primaquine in Azerbijan, Tajikistan, Afghanistan, and DPR Korea to eliminate vivax malaria that is reported here is not widely known. Although detailed information is not available from these more than eight million healthy subjects who received radical curative primaquine courses, the documented experience reported here does provide important information of relevance today both for the efficacy and the safety of primaquine MDA.

When primaquine MDA (in this case, MPPT [24]) achieved high coverage and was associated with adequate vector control activities it was generally highly effective in preventing subsequent vivax malaria and in some circumstances driving the incidence down towards zero. In the converse situation with inadequate coverage and limited vector control activities, it was not effective. Serious toxicity with 14-day primaquine regimens providing total doses of approximately 3.5 mg base/kg was anticipated and prepared for. The interrupted primaquine MPPT regimen, in which there was a three-day, drug-free period after the fourth day, allowed identification of toxicity and intervention if necessary. However reported tolerability of primaquine was generally good (provided the drug was taken with food) and there were surprisingly few reported serious adverse effects, despite high prevalences of G6PD deficiency (which in some areas would have been the severe “Mediterranean variant”) (Table 14). This is not fully explained given the serious haemolysis clearly documented elsewhere in subjects with this variant [43,44].

**Table 14:** Summary of prevalence of side effects for the four MPPT countries

**Country**	**Assessment**	**G6PD prevalence (%)**	**Treated (N)**	**Side effects (%)**	Serious side effects (*)
Geokchai, Azerbaijan	Daily field reports	12-38	30,000	2-4	7 persons
Mirbashir, Azerbaijan	Surveys	5-10	3,000	2.7	0
North Afghanistan	Surveys	0-15	94,000	A few cases	0
Tajikistan	Daily reports	0.8-4	1,374,000	A few cases	0
DPR Korea	Daily field reports	2.9	7,000,000	1.5-4	A few cases

*None of these cases required hospitalization.

In Azerbaijan, which has a very broad range of G6PD deficiency prevalences affecting more than 30% of the population in some areas, MPPT was the only available malaria control option at the time to control or prevent a large scale malaria epidemic. High coverage (of both MPPT standard and interrupted courses) accompanied by well-established, daily, medical supervision and prompt action to mitigate anticipate side effects resulted in the containment of a large-scale malaria epidemic during the 1970s and in the prevention of a malaria epidemic in the 1980s. The intervention was generally well tolerated with very few reports of serious toxicity. In Northern Afghanistan, with moderate levels of G6PD deficiency prevalence, MPPT along with routine vector control activities resulted in a considerable reduction of malaria incidence. Again there was little reported toxicity. In Tajikistan during the 1950s MPPT was deployed as a complementary measure to vector control activities in the residual foci of *P. vivax* malaria with the aim of arresting local malaria transmission. High coverage along with well-functioning malaria surveillance and vector control activities resulted malaria eradication in the republic. Malaria was later reimported and more than 800,000 people in Tajikistan received MPPT during the 1980s-2000s, but MPPT coverage never reached more than 80%. Although in almost all instances MPPT was accompanied by other anti-malaria interventions, these were of very variable quality. Although malaria incidence declined, this reduction was considerably lower than anticipated based on the experiences elsewhere. No serious side effects were reported among the population where the prevalence of G6PD deficiency is approximately 3%. In DPR Korea, which experienced a large-scale malaria epidemic beginning in the late 1990s aggravated by enormous economic hardship, MPPT was considered the only available option to arrest the proliferation of *P. vivax* throughout the country. Vector control interventions were not available and this prompted health authorities to administer MPPT to the population living in areas with high and moderate levels of malaria transmission. A functional public health system with strong community involvement facilitated the achievement of very high treatment coverage. The prevalence of G6PD deficiency is low (<1%) in Korea, and a very low rate of side effects was reported.

This retrospective review of the reported impact of primaquine MPPT in more than eight million people in four different countries inevitably lacks the precision of a prospectively conducted clinical trial. The critical question of the safety of primaquine is not answered definitively. The prevalence and likely severity of G6PD deficiency varied, but certainly the majority of G6PD deficient individuals in Afghanistan would have had the Mediterranean variant [33], which is associated with severe haemolytic reactions following primaquine [43,44]. Severe variants are also likely to have been prevalent in Azerbaijan. Even with the surveillance and reporting system put in place adverse effects may well have been missed, but serious haemolytic toxicity should have been recognized during these interventions, and death from severe anaemia or haemoglobinuric acute renal failure should have been reported. As it was, only a few cases of serious side effects were registered among the millions of treated persons, none of whom required hospitalization, and none died (Table 14). The prevalence of reported adverse effects varied and did not exceed 4%.

## Conclusions

The major benefit of MPPT in the large programmes reviewed here was a considerable reduction of malaria case load, and containment of large-scale vivax malaria epidemics in a relatively short period of time. These programmes show that a reasonably well-organized MPPT on a national (country) scale with the active involvement of health personnel both at the central, provincial and peripheral levels results in a considerable reduction of malaria incidence. MPPT success can be achieved only through the provision of clear instructions and training for all categories of personnel engaged in the treatment, regular efficient supervision, and ready availability of medical support in case of severe side effects. The availability of motivated and disciplined drug distributors and community members resulted in maximal possible coverage of the targeted population in Azerbaijan, North Afghanistan and the DPRK, and to a lesser extent in Tajikistan. Experience demonstrated that cooperation of populations in MPPT was obtained through a vigorous health education campaign with significant involvement of local health and administrative authorities.

The lessons learned from all the MPPT experiences combined are as follows:

1) Efficacious complementary anti-malaria measures are required to achieve a significantly high reduction in malaria incidence;

2) High coverage of between 85-95% (ideally higher than 90%) is necessary for MPPT success;

3) Mobilization of medical staff to document and treat side-effects is essential; and

4) Well-conducted, health education campaigns to motivate the target population to participate are vital.

One of the remarkable achievements of all the reviewed programmes was the very low prevalence of reported side effects, not only among populations with low or moderate levels of G6PD deficiency, but also in areas where severe deficiencies were prevalent. These results support the controlled use of primaquine in MDA strategies for the eradication of vivax malaria provided there is adequate readily available support to manage toxicity.

## Competing interests

The authors declare that they have no competing interests.

## Authors’ contributions

AK conceived of and led the review, assisted by AB and VS. EA, JR, and NW assisted with interpretation and compiling the manuscript. All authors read and approved the final manuscript.
